# Offspring plumage coloration as a condition‐dependent signal in the blue tit

**DOI:** 10.1002/ece3.9787

**Published:** 2023-01-31

**Authors:** Jorge García‐Campa, Wendt Müller, Judith Morales

**Affiliations:** ^1^ Department of Evolutionary Ecology National Museum of Natural Sciences – Spanish National Research Council (CSIC) Madrid Spain; ^2^ Department of Biology, Behavioural Ecology and Ecophysiology Group University of Antwerp Antwerp Belgium

**Keywords:** carotenoids, condition dependence, honest signaling, nestling coloration, quality, sex differences, UV coloration

## Abstract

In many species, offspring display conspicuous coloration already early in life, even though they might be very vulnerable to predation at this stage. However, most attention has been drawn to the conspicuous plumage displayed by adult individuals in a sexual context, while other signaling functions have been explored much less. Here, we investigated whether the yellow breast plumage of blue tit (*Cyanistes caeruleus*) nestlings shows patterns of condition dependence and hence signals individual quality, as has been described for adult birds. During three consecutive breeding seasons, we, therefore, explored the association between nestling body mass and three color components of the yellow breast plumage (i.e., UV chroma, carotenoid chroma, and total brightness), considering both within and among nest effects. Variation in carotenoid chroma was not related to body mass. However, UV chroma and total brightness varied with body mass on an among‐nest level, suggesting that they might signal aspects of genetic quality or parental rearing capacity. Interestingly, we also found a within‐nest effect of body mass on total brightness, suggesting that this is a good candidate for a condition‐dependent signal within the family. Thus, other family members could rely on brightness to adjust their behavioral strategies, such as feeding behavior in parents. Our study thus reveals that certain color components of the yellow breast plumage might signal different aspects of offspring quality, and they might have a correlated signaling value across life‐history stages.

## Graphical abstract

In the present study, we investigated whether the yellow breast plumage of blue tit (*Cyanistes caeruleus*) nestlings shows patterns of condition dependence and hence signals individual quality. We explored the association between nestling body mass and three color components of the yellow breast plumage (i.e., UV chroma, carotenoid chroma, and total brightness) during three consecutive breeding seasons. Our study thus reveals that certain color components of the yellow breast plumage signal different aspects of offspring quality and suggest that they might have a correlated signaling value across life‐history stages.

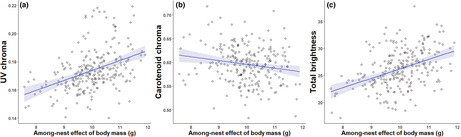

## 1 INTRODUCTION

Coloration is ubiquitous in nature and plays a significant role in animal communication, e.g., in the context of pollination, predator–prey interactions, or mating (Cuthill et al., 2017; Endler & Mappes, 2017; Postema et al., 2022). Colorful traits are often studied in the context of sexual selection where they are seen as handicaps that seem not to increase longevity or fecundity, so they are potentially not favored by natural selection. Conspicuous colorful patches—displayed mainly by males—would rather allow to attract mates or to discourage potential competitors (Andersson, 1994). Hence, colorful ornaments are thought to function as signals of quality to reliably inform conspecifics about, for example, condition (Hill, 2011), immune status (Rodríguez‐Ruiz et al., 2020), or parasitic burden (Megía‐Palma et al., 2016). Then, honesty in signaling traits is achieved through associated costs to produce and maintain them (Andersson, 1994).

However, there are conspicuous colorful traits that are also expressed in sexually non‐mature or even in newborn individuals. In these cases, coloration is displayed in a non‐sexual selection context (West‐Eberhard, 1983) at one of the most vulnerable stages in life. These traits have often been explained as by‐products of selection acting on colouration in adults, at least when both offspring and adults display the same traits (similar to female ornaments, which, initially, were only interpreted as correlated effects of selection in males; see discussion by Amundsen, 2000; see also Doutrelant et al., 2020; West‐Eberhard, 1983). However, given the low‐heritability estimates of certain colorations (Charmantier et al., 2017; Class et al., 2019; Drobniak et al., 2013), offspring coloration may have important signaling functions in itself like signaling quality or need to parents. For example, the occurrence of natal coats in primates—distinct from adult fur—seems to have evolved to solicit greater maternal care (Higley et al., 1987). In birds, American coot (*Fulica americana*) parents preferentially feed the most ornamented offspring (Lyon et al., 1994). Another example is nestling gape coloration, a trait that is adjusted to parent visual performance (Avilés & Soler, 2009) that reflects offspring need (Kilner, 1997; but see Heeb et al., 2003), and that ultimately mediates parental feeding decisions (Gotmark & Ahlstrom, 1997; Kilner, 1997; Saino et al., 2000). There is also some evidence that offspring signals could be perceived by other family members to mediate sib–sib interactions (Dreiss et al., 2016, 2017; Roulin et al., 2000).

Yet, while evidence on the adaptive function of offspring ornaments in the context of parental care is accumulating, little is known about how honesty in these signaling traits can be achieved. The expression of structural ornaments such as plumage colouration requires a substantial investment of resources such as carotenoids, and thus, they can inform parents and other family members (such as siblings and breeding helpers) about individual quality (Caro et al., 2016; Hinde & Kilner, 2007; Morales & Velando, 2013). Like in a sexual selection context, honesty can be achieved if the offspring pay a cost for displaying or maintaining such signaling traits, which prevents cheating (handicap principle; Zahavi, 1977). Therefore, nestling coloration has the potential to evolve as a condition‐dependent signal to which other family members respond (honest signaling models, Fromhage & Henshaw, 2022; Godfray, 1991, 1995; Laidre & Johnstone, 2013). Contrastingly, nestlings may also display color traits that function in a sexual context if these are not replaced before sexual maturity. For example, the tail coloration of blue tit nestlings, unlike other plumage patches, is not replaced after the first year (Peters et al., 2007; Svensson, 1992).

A good model system to study whether conspicuous nestling plumage coloration shows similar patterns of condition dependence in both offspring and adults is the carotenoid‐based coloration of the yellow breast plumage of blue tits (*Cyanistes caeruleus*). Blue tit adults exhibit both UV/blue crown feathers and yellow breast feathers. In adults, UV/blue coloration might function as a sexual signal (Parker, 2013), as it reflects condition (Delhey et al., 2006) and shapes the parental investment of mates (Limbourg et al., 2013a, 2013b). Similarly, yellow breast feathers reliably reflect aspects of individual quality such as parasite burden (del Cerro et al., 2010), parental capacity (García‐Navas et al., 2012), and laying performance (Midamegbe et al., 2013). Furthermore, the UV chroma of adult breast plumage functions as a signal in parental interactions during offspring care (García‐Campa et al., 2022). Blue tit nestlings do not exhibit the UV/blue crown coloration, but there is some evidence that two color parameters of the yellow breast plumage, carotenoid chroma (Johnsen et al., 2003) and UV chroma (Jacot & Kempenaers, 2007; Morales & Velando, 2018), co‐vary with nestling body mass. Moreover, family members rely on nestling UV chroma to adjust their decision rules over parental investment. Concretely, nestlings with experimentally blocked UV color beg more during feeding rates and in sib–sib competitive interactions (Morales & Velando, 2018). In addition, when resources are limited, parents favor chicks with higher UV chroma, thus, presumably those of high quality (García‐Campa et al., 2021; Morales & Velando, 2018). It is possible that the different components of colouration reveal different aspects of individual quality (Candolin, 2004), as they involve different dimensions of avian color perception (Jacot & Kempenaers, 2007): reflectance in the ultraviolet region of the spectrum (UV chroma; a measure of the contribution of UV to the total reflectance), carotenoid‐based reflectance (carotenoid chroma; which reflects the amount of carotenoid pigments deposited in feathers, as it represents the relative reflectance around the absorbance peak of carotenoids), and total reflectance (brightness). Hence, in order to understand the signaling function of yellow breast plumage colouration in blue tit nestlings, it is valuable to investigate the different color components as well as their relationships with condition.

In this study, we first explored the associations between UV‐chroma, carotenoid chroma, and total brightness of blue tit nestling yellow breast feathers. Then, we investigated the relationship of each of the three color components with body mass in three consecutive breeding seasons. We hypothesized that only individuals in good condition (i.e., nestlings with higher body mass) would be able to achieve, in particular, a higher reflectance in the ultraviolet region of the spectrum, as this has been experimentally demonstrated previously (Morales & Velando, 2018). Furthermore, as multiple chicks per nest were measured, we tested whether any effect of body mass on coloration was due to an among‐nest or a within‐nest effect, which, to our knowledge, has not been explored to date. The within‐nest effect allows testing whether chick coloration varies according to within brood differences in body mass, reflecting condition dependence at the nest level. The among‐nest effect in turn would show whether the correlation of body mass and coloration is due to, for instance, genetic effects, parental quality effects, or other (common) environmental effects at the nest level. If nestling yellow plumage functions as a signal in intra‐family interactions, we expect a within‐nest effect of body mass on coloration, since this would allow other family members to assess individual quality relative to other siblings in the nest. The interaction between the within and the among‐nest effect then again would allow testing whether the strength of condition dependence is influenced by brood identity. Unraveling the relationships between offspring colouration and body mass at the among‐nest and within‐nest level is hence of great importance to better understand the signaling function of coloration.

## 2 MATERIALS AND METHODS

### 2.1 General methods

The study was carried out in the locality of Miraflores de la Sierra, Madrid, central Spain (40°48′N, 03°47′W) throughout the breeding seasons of 2017, 2018, and 2019. We studied a blue tit population breeding in nest‐boxes in a deciduous forest, mainly dominated by Pyrenean oak (*Quercus pyrenaica*). At the beginning of the breeding season, we started visiting nest‐boxes every week to record the onset of nest construction. Then, we checked them every 2–3 days to record laying and hatching dates (hatching day = day 0). On days 11 (in 2019) or 12 (in 2017 and 2018), that is, once blue tit nestlings had mostly developed yellow breast feathers (Peters et al., 2007), we measured feather coloration and body mass (see a detailed explanation in *Color measurements* below). On these days, we also took blood samples (in 2017) and 3–5 breast feathers per nestling for molecular sexing (see below). Blue tit nestlings exhibit yellow breast feathers, which they molt about 2 months after fledging, during the post‐juvenile molt (Cramp & Perrins, 1993). Interestingly, blue tit nestlings are sexually dimorphic in the yellow breast feathers at early stages (Johnsen et al., 2003), whereas these differences disappear as adults (Hunt et al., 1998). This pattern is different from other color traits such as the upper‐tail feathers, which are sexually dimorphic both in adults (Hunt et al., 1998) and nestlings (Johnsen et al., 2003). Yellow breast feathers reflect light both in the long‐wave band of the reflectance spectrum (yellow‐to‐red wavelengths between 550 and 700 nm) and in the ultraviolet (UV) region (Shawkey & Hill, 2005).

### 2.2 Color measurements

We measured breast plumage coloration with a portable spectrophotometer (Jazz, OceanOptics©) connected to a Pulsed Xenon Light Source (Jazz PX lamp OceanOptics©). For each nestling, we took three consecutive measurements relative to a white standard and perpendicular to the feather surface, using an external probe fitted with a plastic cylinder to standardize the measuring distance and exclude ambient light. We then obtained the reflectance spectra between 320 and 700 nm using CLR program v 1.1 (Montgomerie, 2009). We excluded the first part of the spectrum (300–320 nm) in order to avoid noisy reflectance values.

We then calculated three objective color parameters: (i) total brightness (i.e., average reflectance between 320 and 700 nm; adapted from Jacot & Kempenaers, 2007), (ii) UV chroma (i.e., reflectance in the UV wave‐band region of the spectrum divided by the total reflectance of the spectrum in the avian visual range (*R*_320–400_/*R*_320–700_); adapted from Johnsen et al., 2003) and (iii) carotenoid chroma (i.e., an estimation of the carotenoid content of yellow breast feathers (*R*_700–_ *R*_450_/R_700_), since carotenoids highly absorb in 450 nm; Shawkey & Hill, 2005). For each color parameter, we then calculated the mean of the three consecutive color measurements sampled per nestling.

We measured the plumage colouration of 1837 nestlings (*n*_2017_ = 672; *n*_2018_ = 639; *n*_2019_ = 526) of which 945 were males and 892 females. We excluded two nestlings in the analysis due to a missing value in the carotenoid chroma and an outlier in the UV chroma (=0.12). Due to the experiments performed in parallel studies, color measurements were taken at the age of 12 days in 2017 and 2018 and 11 days in 2019. On the day of color measurement, we also weighted each nestling to the nearest 0.01 g with an electronic Pesola spring balance.

In 2017, we provided blue tit females with extra lutein pigment prior to and during egg laying (for details, see García‐Campa et al., 2020). However, nestlings of lutein supplemented mothers did not differ in coloration from control nestlings (one‐way ANOVA test; total brightness: *F*_1,670_ = 2.13; *p* = .15; UV chroma: *F*_1,669_ = 0.29; *p* = .59; carotenoid chroma: *F*_1,670_ = 0.02; *p* = .89). In the 2018 season, we reduced the yellow UV chroma of one blue tit parent (indistinctly males and females) at the nest on the second week of nestling age (García‐Campa et al., 2022a). Nonetheless, parental UV chroma did not have an effect on offspring color parameters (one‐way ANOVA test; total brightness: *F*_1,521_ = 0.61; *p* = .44; UV chroma: *F*_1,521_ = 1.20; *p* = .28; carotenoid chroma: *F*_1,521_ = 0.78; *p* = .38). Additionally, in 2017 and 2018, we cross‐fostered full clutches among nests at the end of incubation. Thus, among‐nest differences in the relationship between color and condition could be partly explained by the rearing effects of the foster nest.

### 2.3 Molecular sexing

DNA was extracted from blood samples (in 2017) and 3–5 feather pins (in 2018 and 2019) using the Qiagen DNeasy Blood and Tissue kit (Qiagen Inc.). Sex identification was performed by polymerase chain reaction (PCR) amplification of the CHD‐W and CHD‐Z genes with primers P2 and P8, following Griffiths et al. (1998) with a few modifications. An initial denaturizing step at 94°C for 4 min 30 s was followed by 40 cycles of 94°C during 30 s, 49°C during 45 s and 72°C during 45 s. A final run of 72°C during 10 min completed the program. Amplification was carried out in a total volume of 10 μL. Each PCR sample contained: 2 μL DNA, 0.08 μL Taq polymerase (TaKaRa BIO Inc.), 0.8 μL dNTP 2.5 mM, 0.5 μL of each primer 10 μM, 1 μL of 10x PCR buffer and 5 μL of sterilized distilled water.

### 2.4 Statistical analyses

We used R 4.1.0 (R Core Team, 2020) for statistical analyses. First, to explore how the color parameters were inter‐related, we performed correlations between UV chroma, carotenoid chroma, and brightness both at the individual level and at the nest level (the latter using mean values of color parameters). Second, we fitted three linear mixed models with a normal distribution of errors using the lmer function in the “*lme4*” package (Bates et al., 2015) to determine the relationships between body mass and each of the three color parameters. We assumed normality in all cases after checking the residual plots, given also the robustness of mixed models to violations of normality assumptions (Schielzeth et al., 2020). The models included as fixed effects the average body mass of the brood (=among‐nest effect), the deviation from the average body mass of the brood (=within‐nest effect), and their interaction. We included in addition year (2017, 2018, and 2019), nestling sex, brood size, and the interactions between year and nestling sex, average body mass and year, average body mass and brood size, and nestling sex with the deviation from the average body mass. Backward elimination for nonsignificant interactions (*α* = .05) was used to build the minimal models. We also included nest ID as a random intercept and the interaction between nest ID and the deviation from the average body mass (=within‐nest effect) as a random slope.

## 3 RESULTS

### 3.1 Associations between color parameters

At the individual level, yellow UV chroma of nestlings was positively correlated with total brightness (*r* = .35; *p* < .001; *n* = 1835) and negatively with carotenoid chroma (*r* = −.54; *p* < .001; *n* = 1835). In contrast, there was no relationship between carotenoid chroma and brightness (*r* = −.010; *p* = .70; *n* = 1835). When analyzing the correlations of these color parameters for each sex separately, the results were consistent. Yellow UV chroma of nestlings was positively correlated with total brightness both in males and females (*r*_males_ = .36; *p*_males_ < .001; *n*_males_ = 945; *r*_females_ = .36; *p*_females_ < .001; *n*_females_ = 834) and negatively with carotenoid chroma (*r*_males_ = −.54; *p*_males_ < .001; *n*_males_ = 945; *r*_females_ = −.50; *p*_females_ < .001; *n*_females_ = 834). Moreover, there were no relationships between carotenoid chroma and brightness neither in males and females (*r*_males_ = −.030; *p*_males_ = .36; *n*_males_ = 945; *r*_females_ = −.010; *p*_females_ = .84; *n*_females_ = 834).

**FIGURE 1:**
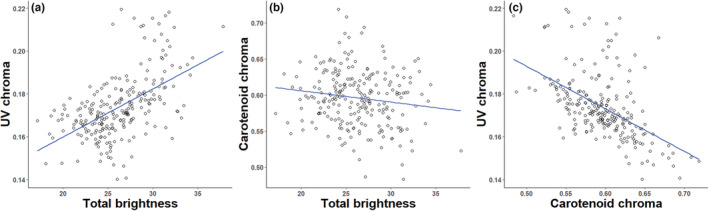
Correlations between yellow breast feather color parameters (mean nest values) measured in blue tit nestlings: (a) UV chroma versus total brightness, (b) carotenoid chroma versus total brightness, and (c) UV chroma versus carotenoid chroma.

When running the correlations of color parameters at the nest level, mean yellow UV chroma was positively correlated with mean total brightness (*r* = .55; *p* < .001; *n* = 234; Figure 1a) and negatively with carotenoid chroma (*r* = −.50; *p* < .001; *n* = 234; Figure 1b). Moreover, the relationship between carotenoid chroma and brightness was marginally significant and negative (*r* = −.14; *p* = .030; *n* = 234; Figure 1c).

### 3.2 Condition dependence

**FIGURE 2:**
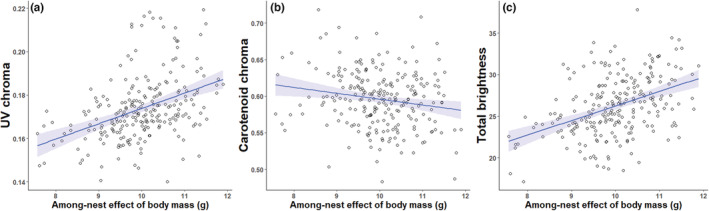
Among‐nest effect of body mass (i.e., average body mass of the brood) on (a) UV chroma, (b) carotenoid chroma, and (c) total brightness of yellow breast feathers measured in blue tit nestlings. Regression lines and ±95% confidence intervals (blue shaded area) are shown.

**FIGURE 3:**
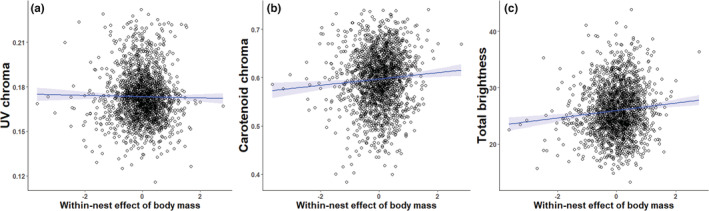
Within‐nest effect of body mass (i.e., individual deviation from the brood average) on (a) UV chroma, (b) carotenoid chroma, (c) total brightness of yellow breast feathers measured in blue tit nestlings. Regression lines and ±95% confidence intervals (blue shaded area) are shown.

We found a significant among‐nest effect of body mass on yellow UV chroma (*F*_1,223.31_ = 8.26; *p* = .0044; Table 1). Broods in which the average nestling mass was higher had higher levels of UV chroma than broods with on average lower nestling body mass (Figure 2a). However, we did not find a significant within‐nest effect of body mass on UV chroma (*F*_1,189.18_ = 2.81; *p* = .096), so that nestlings with lower than average body mass in their brood did not have lower UV chroma than their siblings (Figure 3a; see also Figure S3). Yellow UV chroma was significantly affected by the interaction between year and nestling sex (*F*_2, 1544.02_ = 5.15; *p* = .0059; Figure S1). UV chroma was higher in females than in males in all the seasons (all Post‐Hoc tests: *p* < .001). In females, UV chroma was higher in 2017 than in the other 2 years, and it did not differ between 2018 and 2019 (*p* = .23). The same effect was found for males, but, unlike females, the difference between 2018 and 2019 was almost significant (*p* = .051). The rest of the interactions were not significant (all *p* > .11).

**TABLE 1:** Final mixed models exploring the condition‐dependence of color parameters. Significant effects are marked in bold.

	UV chroma	Brightness	Carotenoid chroma
Intercept	coef. = 139.50 ± 10.26	coef. = 21.02 ± 2.22	coef. = 615.20 ± 34.01
Within‐nest effect of body mass	coef. = 0.68 ± 0.40 *F*_1,189.18_ = 2.81 *p* = .096	coef. = 0.51 ± 0.11 *F*_1,1330.31_ = 21.37 ***p* < .001**	coef. = 2.58 ± 1.85 *F*_1,1159.48_ = 1.96 *p* = .16
Among‐nest effect of body mass	coef. = 2.72 ± 0.95 *F*_1,223.31_ = 8.26 ***p* = .0044**	coef. = 0.47 ± 0.21 *F*_1,226.76_ = 5.22 ***p* = .023**	coef. = −1.46 ± 3.13 *F*_1,225.19_ = 0.22 *p* = .64
Year (2017)	coef. = 11.69 ± 1.09 *F*_2,220.81_ = 57.73 ***p* < .001**	coef. = 3.28 ± 0.24 *F*_1,221.84_ = 98.26 ***p* < .001**	coef. = −14.71 ± 3.59 *F*_1,218.55_ = 9.02 ***p* < .001**
Nestling sex (Males)	coef. = −2.33 ± 0.22 *F*_1,1577.95_ = 109.65 ***p* < .001**	coef. = 0.34 ± 0.07 *F*_1,1625.84_ = 23.05 ***p* < .001**	coef. = 10.26 ± 1.20 *F*_1,1642.16_ = 73.48 ***p* < .001**
Brood size	coef. = 0.70 ± 0.38 *F*_1,231.04_ = 3.39 *p* = .067	coef. = 0.01 ± 0.08 *F*_1,239.62_ = 0.015 *p* = .90	coef. = −0.31 ± 1.27 *F*_1,237.59_ = 0.060 *p* = .81
Year * Nestling sex	coef. = −0.87 ± 0.30 *F*_2,1544.02_ = 5.15 ***p* = .0059**	coef. = 0.32 ± 0.10 *F*_1,1620.65_ = 6.09 ***p* = .0023**	

*Note*: We included nest ID as a random intercept and the interaction between nest ID and the deviation from the average body mass of the brood (=within‐nest effect) as a random slope. Reference levels are “2017” for year effects and “males” for sex effects.

We did not find among‐nest (*F*_1,225.19_ = 0.22; *p* = .64) or within‐nest effects (*F*_1,1159.48_ = 1.96; *p* = .16) of body mass on carotenoid chroma (Table 1; Figure 2b,c; see also Figure S4). Carotenoid chroma significantly differed among years (*F*_2,218.55_ = 9.02; *p* < .001) and sexes (*F*_1,1642.16_ = 73.48; *p* < .001), being higher in 2017 than in the other 2 years (Post‐hoc tests: *p* < .001), and being higher in males than in females. The rest of the interactions were also not significant (all *p* > .63).

Interestingly, we found significant both among‐nest (*F*_1,226.76_ = 5.22; *p* = .023) and within‐nest effects (*F*_1,1330.31_ = 21.37; *p* < .001) of body mass on total brightness (Table 1). Thus, broods with higher than average body mass displayed brighter yellow colourations (Figure 2c), and those nestlings with a higher body mass relative to the average body mass of their brood displayed brighter yellow coloration than their siblings (Figure 3c; see also Figure S5). Total brightness was significantly affected by the interaction between year and nestling sex (*F*_2,1620.65_ = 6.09; *p* = .0023; Figure S2). Total brightness was higher for male nestlings than for female nestlings in years 2017 and 2019, while there was not a significant difference in 2018 (Post‐Hoc test: *p* = .28; Figure S2). All other interactions were not significantly different (all *p* > .30).

## 4 DISCUSSION

Here, we explored whether the conspicuous coloration of blue tits nestlings could signal quality, by investigating its relationships with body mass both at the within‐ and the among‐nest level. Indeed, as has already been suggested (Jacot & Kempenaers, 2007; Johnsen et al., 2003; Morales & Velando, 2018), we found that certain color components of nestling yellow breast feathers reflected body mass. This pattern was particularly relevant for UV chroma and total brightness. Hence, these traits could act as condition‐dependent signals beyond a sexual selection framework, around which most previous work has focused (e.g., del Cerro et al., 2010; Doutrelant et al., 2008, 2012; Ferns & Hinsley, 2008; Ferrer et al., 2015; García‐Navas et al., 2012; Hidalgo‐Garcia, 2006; Midamegbe et al., 2013; Senar et al., 2002). Furthermore, our approach allowed us to explore the potential of the three color components as quality signals both within‐nest and among‐nest contexts.

### 4.1 Associations between color parameters

We found a negative association between carotenoid chroma and UV chroma of yellow breast feathers, in line with previous results in other study populations (Johnsen et al., 2003, 2005). This negative association may be due to the fact that higher amounts of carotenoid pigments in the feathers partly conceal feather structures, which results in lower UV reflectance. Moreover, we found that total brightness was strongly and positively associated with UV chroma, but not with carotenoid chroma. Hence, the overall reflectance of yellow breast coloration indicates to a large extent the reflectance in the UV region of the spectrum. This is particularly relevant in our model system, since UV coloration is more easily perceived by cavity‐nesting birds than carotenoid‐based reflectance (Avilés et al., 2006; Hunt et al., 2003; Wȩgrzyn et al., 2011; Wiebe & Slagsvold, 2009).

### 4.2 Condition dependence: Nestling color as an honest signal of quality

While the color expression of the nestlings' yellow breast feathers showed condition dependency, this effect differed for the three color parameters under study. Furthermore, the contribution of among‐nest effects (which encompass a combination of genetic effects, parental quality effects, or other common environmental effects) and within‐nest effects (reflecting the relative differences in body mass among all the nestlings raised in the same brood) also varied between the color parameters.

We found a significant among‐nest effect of body mass on yellow UV chroma. Broods with higher mean body mass also had higher mean UV chroma. This effect was independent of brood size. Interestingly, UV chroma could reflect genetic effects (e.g., see UV/blue chroma: Charmantier et al., 2017), parental rearing capacity (Senar et al., 2002), food stress (Siefferman & Hill, 2005), or parasite infection (del Cerro et al., 2010; Hill, 2006, 2022), which we cannot separate in our study. However, we did not find differences in UV chroma between nestlings of the same brood (within‐nest effect). This suggests that, at the intra‐brood level, family members might not use UV chroma as a reliable signal of body mass. This was unexpected since we have experimentally demonstrated in the study population that chicks with reduced UV chroma gain less body mass (Morales & Velando, 2018) and that this trait is used as a signal during intra‐family interactions (García‐Antón et al., In press; García‐Campa et al., 2021; Morales & Velando, 2018). Moreover, cavity‐nesting passerines are especially good at detecting changes in UV reflectance (Avilés et al., 2006; Hunt et al., 2003; Wiebe & Slagsvold, 2009), which points to UV chroma as a promising candidate for a signal inside cavities.

However, the current study reveals that total brightness has more potential as a quality signal within nests (as indicated by the significant within‐nest effect of body mass) but that it nonetheless reflects UV chroma. Indeed, brightness is an achromatic component that may be particularly relevant for hole‐nesting bird species that are breeding under low‐light conditions. Accordingly, previous experimental reductions of UV chroma have entailed significant reductions in total brightness (see reflectance spectra in García‐Campa et al., 2021; Morales & Velando, 2018). Had these previous studies aimed at reducing total brightness (and not only UV chroma), they would have likely found stronger effects in intra‐family interactions. Indeed, given the large within‐nest effect of body mass on juvenile brightness, parents could rely on this trait to adjust their feeding strategies within their brood (García‐Campa et al., 2021; Mas & Kölliker, 2011; Morales & Velando, 2018). Therefore, total brightness could have evolved as a condition‐dependent trait to signal nestling quality to other family members.

Furthermore, the fact that the feathers were collected at different ages from the nestlings (i.e., day 11 in 2019 and day 12 in 2017 and 2018) needs to be discussed. Although breast feathers are almost developed at this age, they are still growing (Peters et al., 2007). Thus, coloration could be reflecting feather growth (e.g., see Keyser & Hill, 1999, in blue grosbeaks, *Guiraca caerulea*; Badyaev & Landeen, 2007, in male house finches, *Haemorhous mexicanus*) rather than condition. Nonetheless, we controlled for year in the analyses, which partly accounts for the age difference, and we found that feather coloration in 2017 was overall much different than in the other 2 years. In addition, it is expected that feather growth is faster in nestlings in better condition. Therefore, although we cannot discard that coloration reflected feather growth to a certain extent, this was in turn likely modulated by condition. In addition, our findings show that there were among‐nest effects of nestling body mass on total brightness. Unlike UV chroma, it is less likely that brightness functions as a signal of genetic quality, since it shows low heritability (e.g., see Charmantier et al., 2017 for blue tit adults and Class et al., 2019 for nestlings). However, it may reflect parental quality effects or other (common) environmental effects (e.g., food stress or parasite infection, see above). Surprisingly, there were neither among‐ nor within‐nest effects of nestling body mass on carotenoid chroma, in contrast to a number of previous studies supporting that this color component is condition‐dependent in nestling blue tits (Delhey et al., 2006, 2010; Jacot & Kempenaers, 2007; Johnsen et al., 2003, 2005). One possibility is that differences in the calculation of carotenoid chroma used across studies ((R_UV peak_ – R_450_)/R_UV peak_ in Bleiweiss, 2004; Jacot & Kempenaers, 2007; and (R_700_ – R_450_)/R_700_ in Johnsen et al., 2005) explain this inconsistency. Besides, since this color parameter is strongly dependent on dietary carotenoid availability, it might contain a strong environmental component, which is, however, not captured at the nest level.

### 4.3 Differences between the sexes

We also found a consistent effect of nestling sex on the three color parameters analyzed, in accordance with previous studies (Jacot & Kempenaers, 2007; Johnsen et al., 2003, 2005). Females expressed higher mean values for yellow UV chroma than males, whereas we detected the opposite pattern for carotenoid chroma and brightness (even though UV chroma and brightness are strongly and positively associated). While the blue tit was one of the first species in which a sexual dimorphism in crown UV‐based plumage color was documented, this has not been found in adult yellow breast feathers (Hunt et al., 1998). It is somehow puzzling that the latter trait is dimorphic only in nestlings and juveniles‐ since yellow body feathers are molted a few months after fledging (Cramp & Perrins, 1993; Schoppe, 1977). Thus, parents could potentially rely on both carotenoid‐chroma and total brightness to discriminate offspring sex while adjusting their feeding strategies. Indeed, in other study populations, blue tit males and females receive different food items (García‐Navas et al., 2014) or the total amount of investment (Dickens & Hartley, 2007). In addition, fledging yellow plumage could play a signaling role in family flocks that are formed immediately after fledging (Stenning, 2018) and during social interactions within flocks (Tschirren et al., 2005). These sex‐specific patterns clearly need further study.

## 5 CONCLUSIONS

We show that yellow breast feathers could function as a condition‐dependent signaling trait in nestling blue tits, given the observed associations with body mass. While total brightness and UV chroma (but not carotenoid chroma) seem to reflect genetic or other common environmental effects (=among‐nest effects), total brightness could also act as an honest signal during intra‐family interactions (=within‐nest effect). Intriguingly, plumage color was a sexually dimorphic trait in nestlings, in contrast to the situation in adults, which is at current difficult to explain. It urges further studies to identify possible diverging selective pressures for males and females in the nestling and post‐fledgling periods.

## AUTHOR CONTRIBUTIONS

**Jorge García‐Campa:** Conceptualization (equal); methodology (equal); software (equal); visualization (equal); writing – original draft (equal). **Wendt Müller:** Conceptualization (equal); formal analysis (equal); investigation (equal); methodology (equal); supervision (equal); validation (equal); visualization (equal); writing – original draft (equal). **Judith Morales:** Conceptualization (equal); formal analysis (equal); funding acquisition (equal); investigation (equal); methodology (equal); project administration (equal); resources (equal); supervision (equal); validation (equal); visualization (equal); writing – original draft (equal).

## CONFLICT OF INTEREST STATEMENT

Authors declare that they have no conflict of interest.

## Supporting information


Figures S1–S5.

## Data Availability

García‐Campa et al. (2022b).
