# Supplementary material for: Offspring plumage coloration as a condition‐dependent signal in the blue tit
Source: Ecol Evol. 2023 Jan 31;13(2):e9787. doi: 10.1002/ece3.9787 (PMC9889846; doi:10.1002/ece3.9787)

**APPENDIX**

**Figure S1.** Sex differences in yellow UV chroma of yellow breast feathers measured in blue tit nestlings separated per year (2017, 2018 and 2019). Male and female nestlings differed in the UV chroma during all the breeding seasons (2017, 2018 and 2019; all *P* < 0.001). Values represent means ± SE.


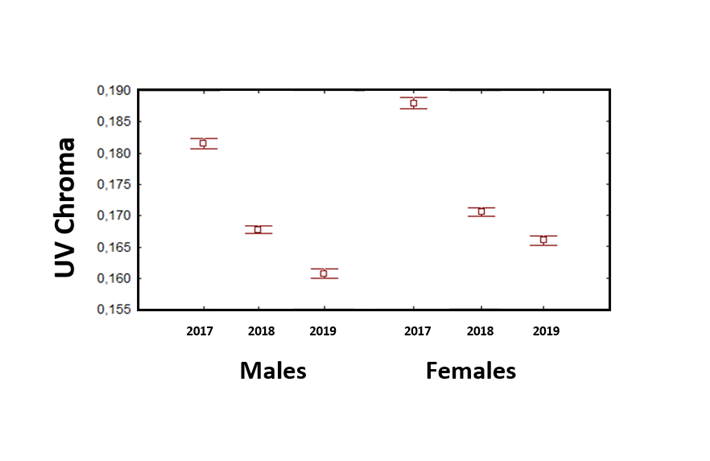


**Figure S2.** Differences in total brightness of yellow breast feathers of blue tit nestlings according to year (2017, 2018 and 2019) and nestling sex of blue tit nestlings. Male and female nestlings differed in the total brightness of yellow breast feathers during 2017 (*P* < 0.001) and 2019(*P* = 0.043). Male and female nestlings did not differ in their total brightness in 2018 (*P* = 0.28). Values are (mean ± SE).


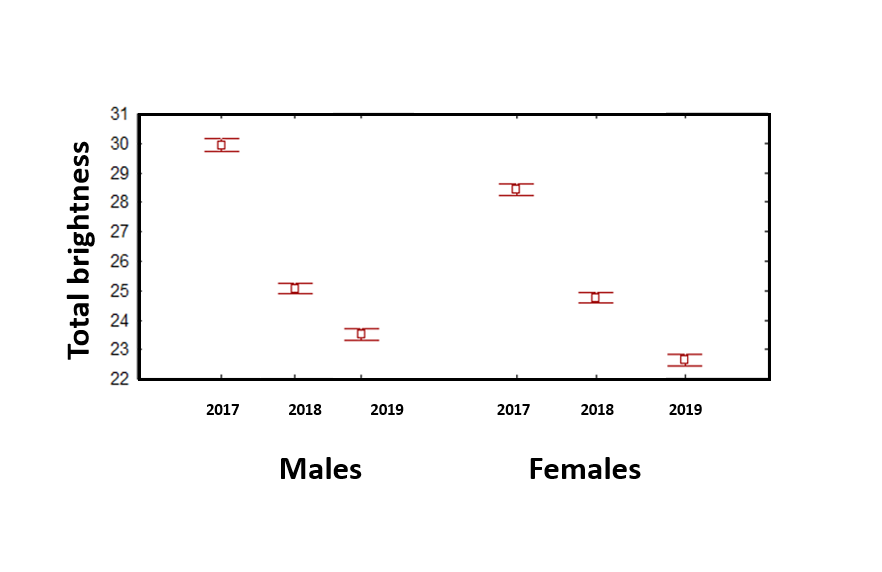


**Figure S3.** The effect of blue tit nestling body mass on UV chroma of yellow breast feathers in each breeding season (i.e., 2017, 2018 and 2019). Lines represent regression lines corresponding to the effect of body mass on UV chroma within broods. Different colours represent different broods.


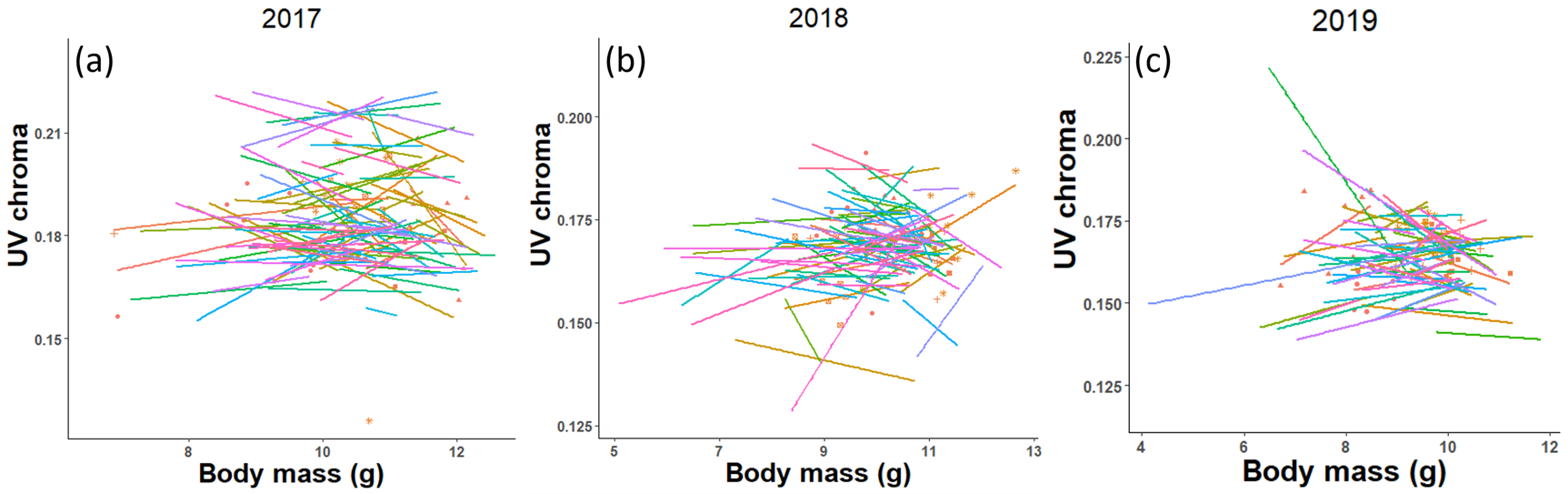


**Figure S4.** The effect of blue tit nestling body mass on carotenoid chroma of yellow breast feathers in each breeding season (i.e., 2017, 2018 and 2019). Lines represent regression lines corresponding to the effect of body mass on carotenoid chroma within broods. Different colours represent different broods.


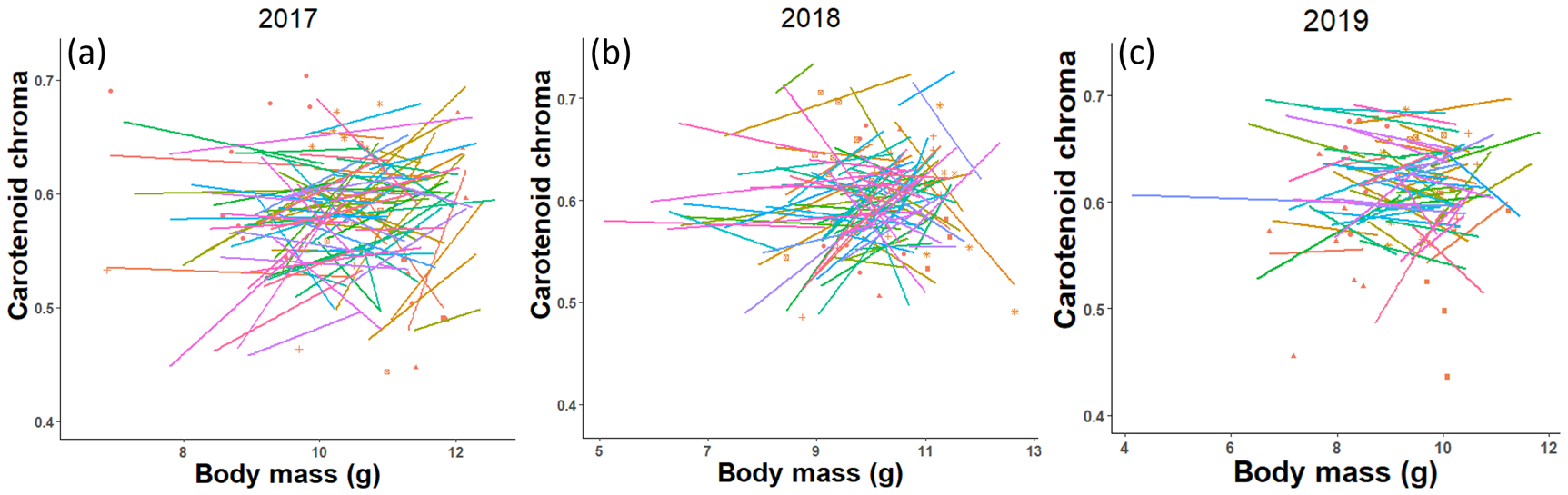


**Figure S5.** The effect of blue tit nestling body mass on total brightness of yellow breast feathers in each breeding season (i.e., 2017, 2018 and 2019). Lines represent regression lines corresponding to the effect of body mass on total brightness within broods. Different colours represent different broods.


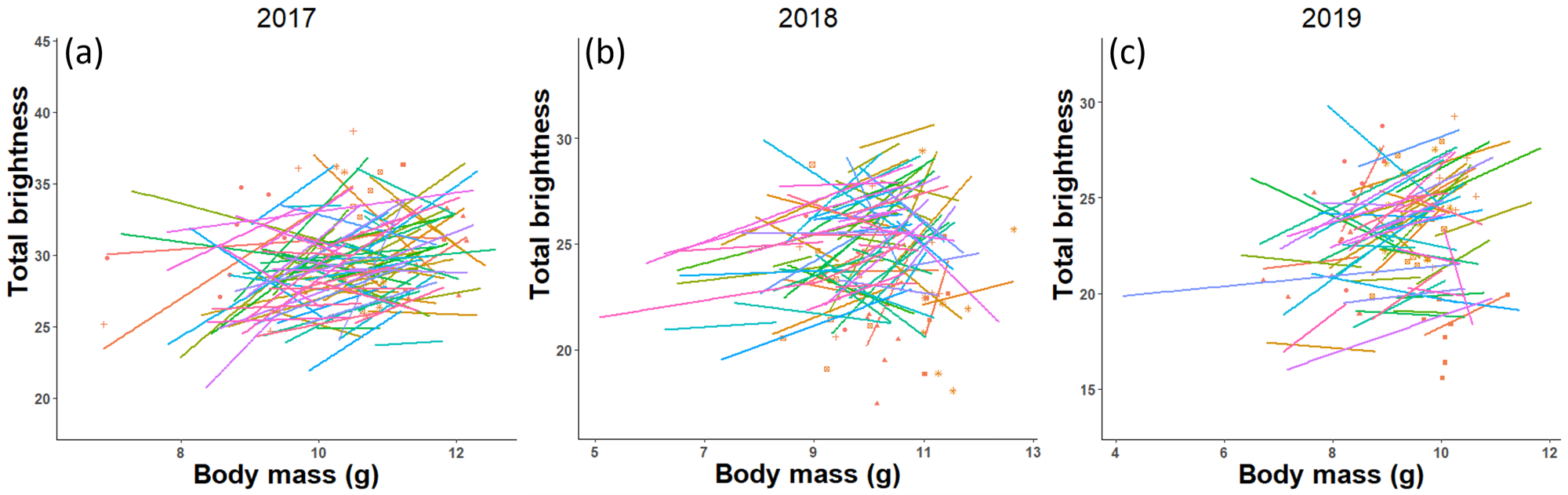

Supplement: Supplementary file 1 — Figures S1–S5. [file ECE3-13-e9787-s001.docx]
